# Comparability of Titers of Antibodies against Seasonal Influenza Virus Strains as Determined by Hemagglutination Inhibition and Microneutralization Assays

**DOI:** 10.1128/JCM.00750-20

**Published:** 2020-08-24

**Authors:** Marten Heeringa, Brett Leav, Igor Smolenov, Giuseppe Palladino, Leah Isakov, Vincent Matassa

**Affiliations:** a Clinical Development, Seqirus Netherlands B.V., Amsterdam, the Netherlands; b Clinical Development, Seqirus Inc., Cambridge, Massachusetts, USA; c Research and Development, Seqirus Australia Pty Ltd., Parkville, Victoria, Australia; Cepheid

**Keywords:** HI assay, hemagglutination inhibition, influenza, MN assay, microneutralization inhibition

## Abstract

We compared titers of antibodies against A/H1N1, A/H3N2, and B influenza virus strains collected pre- and postvaccination using hemagglutination inhibition (HI) and microneutralization (MN) assays and data from two vaccine trials: study 1, performed with a cell-grown trivalent influenza vaccine (TIVc) using cell-grown target virus in both assays, and study 2, performed with an egg-grown adjuvanted quadrivalent influenza vaccine (aQIVe) using egg-grown target virus. The relationships between HI- and MN-derived log-transformed titers were examined using different statistical techniques. Deming regression analyses showed point estimates for slopes generally close to 1 across studies and strains. The slope of regression was closest to 1 for A/H3N2 strain when either cell- or egg-grown viral target virus was used. Bland-Altman plots indicated a very small percentage of results outside 2 and 3 standard deviations. The magnitudes and directions of differences between titers in the two assays varied by study and strain. Mean differences favored the MN assay for A/H1N1 and B strains in study 1, whereas the titers determined by HI were higher than those determined by MN against the A/H3N2 strain. In study 2, mean differences favored the MN assay for A/H3N2 and B strains. Overall, the directions and magnitudes of the mean differences were similar between the two vaccines. The concordance correlation coefficient values ranged from 0.74 (A/H1N1 strain, study 1) to 0.97 (A/H3N2 strain, study 1). The comparative analysis demonstrates an overall strong positive correlation between the HI and MN assays. These data support the use of the MN assay to quantify the immune response of influenza vaccines in clinical studies, particularly for the A/H3N2 strain.

## Abstract (precis)

We compared titers of antibodies against A/H1N1, A/H3N2, and B influenza virus strains collected pre- and postvaccination using hemagglutination inhibition (HI) and microneutralization (MN) assays and data from two vaccine trials: study 1, performed with a cell-grown trivalent influenza vaccine (TIVc) using cell-grown target virus in both assays, and study 2, performed with an egg-grown adjuvanted quadrivalent influenza vaccine (aQIVe) using egg-grown target virus. The relationships between HI- and MN-derived log-transformed titers were examined using different statistical techniques.

## INTRODUCTION

The HI assay is a widely used serological technique considered by many regulatory authorities to be likely to predict clinical benefit of vaccines (1). It is fast, cost-effective, and relatively easy to perform and is considered the gold standard immunological outcome measure. This assay measures the effect of the antibodies that are used to prevent the binding of viral hemagglutinin (HA) to sialic acid residues on the surface of erythrocytes. The HI titer is expressed as the reciprocal of the highest serum dilution that shows complete inhibition of erythrocyte agglutination (2). Recently, the circulating strains of A/H3N2 influenza virus have displayed a phenomenon of reduced hemagglutination activity. The mechanistic explanation for this appears to be the presence of amino acid substitutions in the receptor binding site of the HA molecule (3). This mutation poses a technical challenge for the use of the HI assay for both antigenic analysis and measurement of serologic immune response to vaccination against the A/H3N2 strain; a concern most acute for cell-derived vaccines because of their similarity to the circulating virus (4, 5).

An alternative to the HI assay, the microneutralization (MN) assay, has been used for many years to measure humoral immune responses to influenza and more recently to antigenically characterize influenza viruses (3). The MN assay has several advantages compared to the HI assay. As a functional assay, it represents a more mechanistically relevant estimation of protection by measuring the concentration of antibodies needed to prevent infection of a eukaryotic cell and block the cytopathic effects of a virus *in vitro* (6–8). In addition, the mutations in the A/H3N2 hemagglutinin do not appear to affect the capacity of the virus to infect mammalian cells in culture; therefore, the MN assay can be used instead of the HI assay for antigenic typing and to quantify antibody responses of A/H3N2 vaccine strains regardless of egg or cell derivation and propagation (6). Objective measures of quantification, such as those obtained with an enzyme-linked immunosorbent assay (ELISA) plate reader, can be readily applied to the MN assay, in contrast to the observer-dependent measures of the HI assay. These features of the MN assay provide several distinct advantages over the HI assay. However, potential disadvantages may include increased cost and time.

Previous studies directly comparing HI and MN assays were performed on the basis of relatively low numbers of samples with limited selections of influenza vaccines and under laboratory testing conditions (7, 9–12). The study described here was designed to address these gaps by comparing HI and MN antibody titers by the use of paired testing as obtained from a large number of serum samples against A/H1N1, A/H3N2, and B strains using vaccines manufactured under conditions of egg- and cell-derived platforms and laboratory test conditions.

## MATERIALS AND METHODS

### Participants and serum collection.

Sera were collected from two randomized controlled clinical studies, both of which were approved by the relevant Institutional Review Boards or Ethics Committees before study start. In both studies, blood was drawn before vaccination and 3 weeks after vaccination, for evaluation of both HI and MN titers. Study 1 was a phase I/II, randomized, multicenter immunogenicity and safety study of cell-derived trivalent influenza vaccine (TIVc) and egg-based trivalent influenza vaccine (TIVe) (Fluzone; Sanofi, Paris, France) in subjects 6 months through 4 years of age (ClinicalTrials registration no. NCT02035696). The study was conducted in Finland, the Philippines, Thailand, and the United States from December 2013 to December 2014. Participants were randomly assigned to receive one of three dose levels of TIVc (hemagglutinin content of 22.5 to 67.5 μg per dose; *n* = 507) or a single dose of TIVe (22.5 to 45 μg hemagglutinin per dose; *n* = 164). None of the subjects had been vaccinated previously; therefore, all of the subjects received two vaccinations at time points 4 weeks apart. Blood specimens were drawn before first vaccination and at 3 weeks after the second vaccination. The cell-based vaccine used in study 1 was manufactured using seed virus passaged in egg and then grown in cells, for all strains. The egg-based vaccine was manufactured using seed virus passaged in egg and grown in eggs. Table 1 lists the vaccine and target virus strains used in study 1.

**TABLE 1:** Vaccine strains and target virus strains used in studies 1 and 2^*a*^

Study and virus	Vaccine strain		Target virus strain and assay	
	TIVc	TIVe	HI	MN
1				
A/H1N1	A/Brisbane/10/2010 (wild type) (egg-seed/cell-grown)	A/Brisbane/10/2010 (wild type) (egg-seed/egg-grown)	A/Brisbane/10/2010 (egg seed/cell-grown)	A/Brisbane/10/2010 (wild type) (egg-seed/egg-grown)
A/H3N2	NYMC X-223 (reassortant) derived from A/Texas/50/2012 (egg-seed/cell-grown)	NYMC X-223A (reassortant) derived from A/Texas/50/2012 (egg-seed/egg-grown)	A/Texas/50/2012 (X-223A) (egg-seed/cell-grown)	NYMC X-223 (reassortant) derived from A/Texas/50/2012 (egg-seed/cell-grown)
B	B/Massachusetts/2/2012 (wild type) (egg-seed/cell-grown)	B/Massachusetts/2/2012 (wild type) (egg-seed/egg-grown)	B/Massachusetts/02/2012 (egg-seed/cell-grown)	B/Massachusetts/2/2012 (wild type) (egg-seed/egg-grown)

2	aQIV	QIV	HI	MN
A/H3N2	A/Texas/50/2012 (egg-seed/egg-grown)	A/Texas/50/2012 (egg-seed/egg-grown)	A/Texas/50/2012 (X-223) (egg-seed/egg-grown)	A/Texas/50/2012 (X-223) (egg-seed/egg-grown)
B	B/Massachusetts/2/2012 (egg-seed/egg-grown)	B/Massachusetts/2/2012 (egg-seed/egg-grown)	B/Massachusetts/2/2012 (BX-51B) (egg-seed/egg-grown)	B/Massachusetts/2/2012 (BX-51B) (egg-seed/egg-grown)

a Abbreviations: aQIV, adjuvanted quadrivalent influenza vaccine; HI, hemagglutination inhibition; MN, microneutralization; QIV, nonadjuvanted quadrivalent influenza vaccine; TIVc, cell-derived trivalent influenza vaccine; TIVe, egg-derived trivalent influenza vaccine. Cell-grown, grown in mammalian cells (Madin-Darby canine kidney [MDCK] cells) in liquid culture as a host for the growing influenza virus. Cell-seed: candidate vaccine virus passaged in cells, provided by WHO GISRS Partner to private sector. Egg-grown, grown in fertilized hen eggs, as a host for the growing influenza virus. Egg-seed: candidate vaccine virus passaged in eggs, provided by WHO GISRS Partner to private sector.

Study 2 was a phase III, randomized, multicenter immunogenicity, safety, and efficacy study of an adjuvanted quadrivalent influenza vaccine (aQIV) and a nonadjuvanted comparator influenza vaccine in subjects 6 months through 6 years of age (ClinicalTrials registration no. NCT01964989); the trial design and results were published elsewhere (13). Study 2 was conducted in Canada, Finland, Italy, Mexico, the Philippines, Poland, Puerto Rico, Spain, Taiwan, Thailand, and the United States over two seasons from November 2013 to April 2016. The comparator was a nonadjuvanted trivalent influenza vaccine (TIV) in the first season and quadrivalent influenza vaccine (QIV) in the second season. Subjects were randomly assigned to receive either aQIV (HA content of 30 to 60 μg per dose; *n* = 5,352) or nonadjuvanted comparator influenza vaccine (22.5 to 45 μg hemagglutinin per dose for TIV and 30 to 60 μg hemagglutinin per dose for QIV; *n* = 5,292). Sampling for immunogenicity was conducted in a subset of 2,886 subjects, among which 1,481 subjects received aQIV and 1,405 subjects received comparator vaccine. Depending on age and vaccination history, subjects received either one or two doses at time points 4 weeks apart. Blood specimens were drawn before the first vaccination and 3 weeks after the second vaccination. All vaccines from study 2 were egg-grown (Table 1). There were no MN data available for A/H1N1; thus, that strain from study 2 is not included in the present study results.

Study 2 evaluated egg-based vaccines, manufactured using seed virus passage in egg and grown in eggs. The vaccine strains and target virus used in study 2 are listed in Table 1.

### Laboratory testing.

The HI assay used to measure immunogenicity in study 1 was conducted at the former Novartis Clinical Serology Laboratory in Marburg, Germany. The HI assay used in study 2 was conducted at Viroclinics in Rotterdam, the Netherlands. Target virus strains for both studies are listed in Table 1. In study 1, the HI assay was performed according to the WHO method (14), as follows: In 96-well, V-bottom plates (25 μl/well), heat-inactivated sera treated with receptor-destroying enzyme were serially diluted 2-fold, starting from 1:10 dilution, in phosphate-buffered saline. Virus was added (4 hemagglutinin units per well in 25 μl) and incubated at room temperature for 60 min. After incubation, 50 μl/well of turkey erythrocyte solution (0.5% [vol/vol] in phosphate-buffered saline) was added, and the plates were further incubated at room temperature for 60 min, when inhibition of hemagglutination was determined by visual inspection. In study 2, the protocol was modified as follows: In 96-well, U-bottom plates (50 μl/well), heat-inactivated sera treated with receptor-destroying enzyme were serially diluted 2-fold, starting from 1:20 dilution, in phosphate-buffered saline. Virus was added (4 hemagglutinin units per well in 25 μl) and incubated at 37°C for 30 min. After incubation, 25 μl/well of turkey erythrocyte solution (1% [vol/vol] in phosphate-buffered saline) was added, and the plates were further incubated at 4°C for 60 min, when inhibition of hemagglutination was determined by visual inspection.

The MN assay used in study 1 was conducted at Southern Research (Birmingham, AL, USA). The MN assay used to measure immunogenicity in study 2 was conducted at Viroclinics in Rotterdam, the Netherlands (see Table 1 for a list of test strains). The MN assay (14) was performed as follows. In 96-well, flat-bottom plates (50 μl/well), heat-inactivated sera were serially diluted 2-fold, starting from 1:10 dilution, in Dulbecco’s modified Eagle medium with bovine serum albumin. Equal volumes of virus, diluted to 100 TCID_50_ (median tissue culture infectious doses) per well in medium with l-(tosylamido-2-phenyl) ethyl chloromethyl ketone–trypsin, were added, and plates were incubated at 37°C for 60 to 120 min. After incubation, Madin-Darby canine kidney cells were added at 1.5−E4 cells/well in 100 μl of medium, and the plates were further incubated for 18 to 21 h at 37°C in 5% CO_2_. At that time, cells were fixed with 80% acetone for 15 min at room temperature. After washing, the primary staining antibodies (mouse monoclonal anti-influenza A virus or B virus nucleoprotein antibodies) were added and incubated at room temperature for 60 min. After additional washing, the secondary staining antibodies (goat anti-mouse immunoglobulin G conjugated with horseradish peroxidase antibodies) were added and incubated at room temperature for an additional 60 min. After the final wash, the enzyme substrate was added and incubated at room temperature for 15 to 20 min and the reaction stopped with a stop solution. Absorbance was determined using a spectrophotometer, and the 50% virus neutralization (NT) titer of each serum was determined.

### Statistical analysis.

Analyses were conducted in a pairwise manner, wherein each component in a pair was compared with the other component. For each pair, the analyses were done by strain and study (study 1 with 3 seasonal strains and study 2 with 2 seasonal strains). The primary analysis of the numerical data was performed by assigning all titer values below the lower limit of quantification (which was <10) a value of 5. The log_2_-transformed titer values were used for all analyses of numerical results.

Deming regression analysis was conducted to determine the relationship between HI- and MN-derived log-transformed titers. Deming regression appropriately allows for variability in the *x* variable (HI) and the *y* variable (MN) (15, 16). Slope and intercept estimates and their 2-sided 95% confidence intervals (CIs) were determined. Scatterplots (with the associated regression lines) of the HI- and MN-derived log-transformed titers superimposed with the line of identity (*y *=* x*) were also determined. Deming regression lines were fitted for the numerical data with imputation and without imputation. Data with imputation were used for the primary statistical analysis. Data without imputation of a lower limit of quantification, which were used for the sensitivity analysis, were consistent with the results of the primary analysis and are not presented here.

Bland-Altman plots were generated to describe agreement between two quantitative measurements by constructing limits of agreement. These statistical limits are calculated by using the mean and the standard deviation (SD) of the differences between two measurements. The resulting graph is a scatterplot *xy*, in which the *y* axis data represent the differences between the two paired HI- and MN-derived log-transformed titers (log_2_ HI – log_2_ MN) and the *x* axis data represent averages of these measures ([log_2_ HI + log_2_ MN]/2). The mean difference ±1.96 SD of the difference defines the limits of agreement (17). Therefore, lines representing a difference of 0 (no bias) and the observed mean difference were added to each plot to demonstrate potential bias and lines delineating ±2 SD and ±3 SD have been added to represent the limits of agreement. At least 95% of paired measurements were expected to lie within ± 2 SD of the mean difference, and 99.7% of the paired data points were expected to lie within ± 3 SD of the mean difference.

Lin’s concordance correlation coefficients (CCC) and the precision and accuracy components of the CCC were determined (18). The CCC is defined as CCC = precision times accuracy, where precision is the Pearson correlation coefficient, a measure of how far each observation deviates from the best-fitted line, and accuracy is a bias correction factor that measures how far the best-fitted line deviates from the 45° line through the origin.

In the pediatric population, HI titer thresholds of 1:110 against A/H3N2 and 1:40 against B strains have previously been correlated with a 50% clinical protection rate against influenza (19–21). These HI threshold titers were used to predict corresponding MN thresholds by applying slope and intercept estimates from Deming regression analyses.

## RESULTS

The analysis used data from 3,983 subjects and 3 seasonal strains from study 1 and 4,167 subjects and 2 seasonal strains from study 2 (Table 2).

**TABLE 2:** Number of subjects with paired results available for quantitative analyses for each strain and each study^*a*^

Strain	No. of subjected with indicated results					
	Study 1			Study 2		
	TIVc	TIVe	Total	aQIV	QIV	Total
A/H1N1	1,002	323	1,325	ND	ND	ND
A/H3N2	1,004	325	1,329	1,036	1,020	2,056
B	1,004	325	1,329	1,070	1,041	2,111

a Abbreviations: aQIV, adjuvanted quadrivalent influenza vaccine; ND, not determined; QIV, nonadjuvanted quadrivalent influenza vaccine; TIVc, cell-derived trivalent influenza vaccine; TIVe, egg-derived trivalent influenza vaccine.

**FIG 1:**
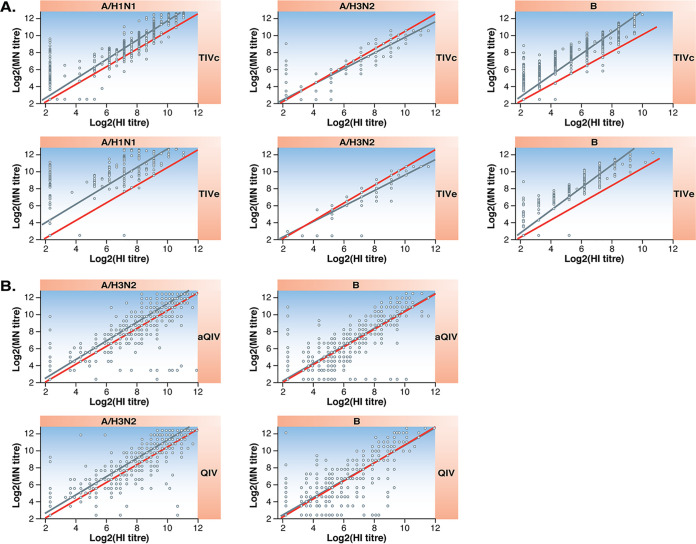
Deming regression scatterplots of the paired results (log_2_-transformed HI titer versus the log_2_-transformed MN titer). Red lines indicate perfect correlation between HI and MN titers. Blue lines represent the Deming regression line. (A) Study 1 (TIVc versus TIVe). (B) Study 2 (aQIV versus QIV).

The Deming regression results appear in Fig. 1 and Table 3. Except for the B strain in study 1, the point estimates for slopes ranged from 0.89 to 1.27. The point estimate of slope was greater than 1 in all cases except for A/H3N2 in study 1. The regression line deviated from the perfect agreement line for TIVe A/H1N1, the TIVe B strain, and the TIVc B strain. However, in all cases, the slopes were similar for the two vaccines.

**TABLE 3:** Slope and intercept estimates from Deming regression^*a*^

Study and vaccine	Strain	Slope (95% CI)	Intercept (95% CI)
1			
TIVc	A/H1N1	1.14 (1.13 to 1.16)	−0.04 (−0.15 to 0.09)
	A/H3N2	0.91 (0.90 to 0.92)	0.29 (0.20 to 0.37)
	B	1.40 (1.38 to 1.43)	−0.55 (−0.65 to −0.45)
TIVe	A/H1N1	1.27 (1.21 to 1.32)	0.48 (0.08 to 0.89)
	A/H3N2	0.89 (0.87 to 0.91)	0.28 (0.11 to 0.45)
	B	1.41 (1.36 to 1.47)	−0.61 (−0.82 to −0.40)

2			
aQIV	A/H3N2	1.18 (1.14 to 1.21)	−0.73 (−1.05 to −0.42)
	B	1.16 (1.13 to 1.19)	−0.77 (−0.95 to −0.58)
QIV	A/H3N2	1.13 (1.10 to 1.16)	−0.31 (−0.55 to −0.07)
	B	1.16 (1.13 to 1.19)	−0.63 (−0.80 to −0.46)

a Abbreviations: aQIV, adjuvanted quadrivalent influenza vaccine; CI, confidence interval; QIV, nonadjuvanted quadrivalent influenza vaccine; TIVc, cell-derived trivalent influenza vaccine; TIVe, egg-derived trivalent influenza vaccine.

**FIG 2:**
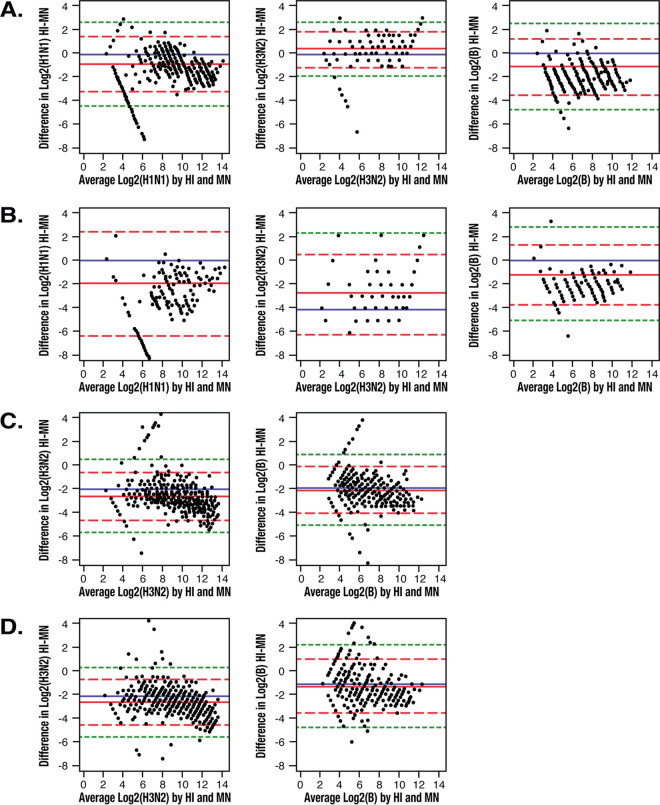
Bland-Altman plots of difference in log_2_ (titer) hemagglutination inhibition (HI) assay minus the microneutralization (MN) assay against the average. The blue line represents a difference of 0 (no difference). The red solid line indicates the mean difference (equivalent to a systematic shift). The red dashed lines represent the mean differences ±2 standard deviations (SDs; the limits of acceptance) within which most of the pair measurements are expected to lie. The green dotted lines represent ±3 SDs from the mean difference. (A) Study 1 versus TIVc. (B) Study 1 versus TIVe. (C) Study 2 versus aQIV. (D) Study 2 versus QIV.

Bland-Altman plots representing the difference (HI – MN) against the average over the respective pairs are presented in Fig. 2. A low percentage of results were outside the ±2 SD range or the ±3 SD range (Table 4). Overall, the directions and magnitudes of the mean differences were similar between the two vaccines for comparisons within study and strain. The mean differences favored the MN assay for the A/H1N1 and B strains in study 1, while the HI assay resulted in higher titers than the MN assay against the A/H3N2 strain. In study 2, the mean differences favored the MN assay for the A/H3N2 and B strains.

**TABLE 4:** Summary statistics for concordance correlation coefficient and its precision and accuracy components^*a*^

Study and vaccine	Strain	Concordance correlation coefficient^*b*^ (95% CI)	Precision coefficient^*c*^ (95% CI)	Accuracy coefficient^*d*^ (95% CI)
1				
TIVc	A/H1N1	0.93 (0.92 to 0.93)	0.96 (0.96 to 0.96)	0.97 (0.96 to 0.97)
	A/H3N2	0.97 (0.97 to 0.97)	0.98 (0.98 to 0.98)	0.99 (0.99 to 0.99)
	B	0.82 (0.80 to 0.83)	0.94 (0.93 to 0.94)	0.87 (0.86 to 0.88)
TIVe	A/H1N1	0.74 (0.70 to 0.77)	0.85 (0.83 to 0.88)	0.87 (0.84 to 0.89)
	A/H3N2	0.95 (0.95 to 0.96)	0.97 (0.97 to 0.98)	0.98 (0.97 to 0.98)
	B	0.82 (0.79 to 0.84)	0.94 (0.93 to 0.95)	0.87 (0.85 to 0.88)

2				
aQIV	A/H3N2	0.87 (0.86 to 0.89)	0.90 (0.89 to 0.91)	0.96 (0.96 to 0.97)
	B	0.86 (0.85 to 0.88)	0.87 (0.86 to 0.88)	0.99 (0.99 to 0.99)
QIV	A/H3N2	0.89 (0.88 to 0.90)	0.92 (0.91 to 0.93)	0.97 (0.96 to 0.97)
	B	0.85 (0.83 to 0.86)	0.85 (0.84 to 0.87)	0.99 (0.99 to 0.99)

a Abbreviations: aQIV, adjuvanted quadrivalent influenza vaccine; CI, confidence interval; QIV, nonadjuvanted quadrivalent influenza vaccine; TIVc, cell-derived trivalent influenza vaccine; TIVe, egg-derived trivalent influenza vaccine.

b The concordance correlation coefficient is a measure of agreement along the identity line, calculated as precision multiplied by accuracy.

c The precision component is equivalent to the Pearson correlation coefficient, a measure of the deviation from the best-fitted line.

d Accuracy is a correction factor that measures how far the best-fitted line deviates from the line of identity.

As shown in Table 4, overall the CCC ranged from 0.74 (A/H1N1 strain, study 1) to 0.97 (A/H3N2 strain, study 1). For all strains and vaccines, the Pearson’s correlation coefficient (precision component of the CCC) was 0.85 to 0.98. The strong correlation coefficients of MN and HI across strains and vaccines indicate a high interassay association between the two assays for the seasonal strains. The accuracy coefficient was 0.87 to 0.99 across all strains.

Based on slope and intercept estimates from Deming regression, an HI titer of 1:40 was predicted to correspond to MN titers of 65 to 151 (A/H1N1), 32 to 52 (A/H3N2), and 42 to 119. An HI titer of 1:110, previously associated with protection in children for A/H3N2, corresponded to MN titers of 207 to 546, (A/H1N1), 80 to 163 (A/H3N2), and 137 to 495 (B) (Table 5).

**TABLE 5:** Predicted MN titers as estimates of protective effectiveness based on slope and intercept estimates from Deming regression^*a*^

Vaccine	Strain	Titer		
		Predicted MN (at HI 1:10)	Predicted MN (at HI 1:40)	Predicted MN (at HI 1:110)
TIVc	A/H1N1	13	65	207
	A/H3N2	10	35	88
	B	17	119	492

TIVe	A/H1N1	26	151	546
	A/H3N2	9	32	80
	B	17	119	495

aQIV	A/H3N2	9	47	155
	B	8	42	137

QIV	A/H3N2	11	52	163
	B	9	47	151

a Abbreviations: aQIV, adjuvanted quadrivalent influenza vaccine; MN, microneutralization; QIV, nonadjuvanted quadrivalent influenza vaccine; TIVc, cell-derived trivalent influenza vaccine; TIVe, egg-derived trivalent influenza vaccine.

## DISCUSSION

The HI assay has traditionally been used for characterization of immune response after influenza vaccination (1). However, recent mutations in influenza virus hemagglutinin prevent A/H3N2 strains from agglutinating chicken or turkey erythrocytes, which poses a significant technical challenge for the use of the HI assay (22, 23). These limitations become particularly important in evaluations of cell-derived influenza vaccines, because the candidate vaccine viruses used in manufacture more closely resemble wild-type viruses than egg-based vaccine strains, where egg-induced mutations often increase the hemagglutination activity of viruses with naturally low hemagglutination (24–27). The MN assay is less affected by genetic changes to hemagglutinin that affect agglutination, which has prompted renewed interest in the use of MN to characterize the immune response to influenza vaccination (28).

This study demonstrated a strong positive correlation between HI and MN assays (Pearson’s *r *= 0.85 to 0.98) across strains and vaccines, which indicates a high interassay association between the two assays for the seasonal strain. The correlation was particularly high for the A/H3N2 strains regardless of the vaccine used in the clinical study. Furthermore, Deming regression analysis showed that HI and MN titers were highly correlated across the two trials, with slopes of regression close to 1. This finding was consistent across the trials, vaccines (whether cell-grown or egg-grown, adjuvanted or nonadjuvanted), and influenza virus strains used in the assays. The slope of the regression was closest to 1.0 in comparisons of the MN and HI results for the A/H3N2 strains in both trials.

Previous studies correlated with our observation of a strong positive correlation of HI and MN assays. In a study involving 450 human serum samples, strongly positive Pearson’s correlations between HI and MN assays were shown for seasonal A strains (A/H1N1 A/California/7/2009, *r *= 0.81; A/H3N2 A/Texas/50/2012, *r *= 0.84) and B strains (B/Brisbane/60/2008 Victoria lineage, *r *= 0.71; B/Massachusetts/02/2012 Yamagata lineage, *r *= 0.62) (9). In another study using the A/California/7/2009 strain isolated from 87 confirmed cases, a strong positive correlation (Spearman’s rank correlation, *r* = 0.84) was noted between HI and MN titers (10). Other data demonstrated a similar correlation with other influenza virus strains. In a study involving 732 children, the Spearman rank correlation between HI and MN for A/Brisbane/10/2007 (A/H3N2) was 0.50 (*P < *0.01) (11), and another study enrolling 656 children demonstrated significant correlation between HI and MN for the same strain (β = 0.389, *P < *0.0001) as well as A/Brisbane/59/2007 (A/H1N1; β = 0.588, *P < *0.0001) (7). A smaller study using sera from 151 subjects and 12 historic and recently circulating strains of seasonal influenza A virus demonstrated a high positive mean correlation between HI and virus neutralization (NT) assays (Spearman’s rank correlation, ρ = 0.86) across all strains. Correlation was highest within subtypes and within close proximity in time, as correspondence changed with age. In this study, HI = 20 corresponded to NT = 10, and HI = 40 corresponded to NT = 20 (12). This finding confirmed the practice of considering an HI titer of 40 to correspond with a gold standard of NT = 20 for influenza virus overall as well as for A/H3N2 strains (29). Consistent with these human studies, early animal studies showed that antibody concentrations specific for equine influenza virus measured using the HI assay are highly correlated with the concentrations detected using a virus neutralization assay (30, 31).

The present *post hoc* analysis has some limitations. The data set consisted of two study cohorts, and different serology laboratories were used for the HI and MN assays within and between studies. In addition, a variety of influenza vaccines and assay target viruses were involved. Study 1 compared a cell-grown to an egg-grown trivalent influenza vaccine using a cell-grown reagent (against A/H3N2) in both assays, while study 2 compared an adjuvanted quadrivalent to a nonadjuvanted quadrivalent vaccine (both egg based) using egg-grown target virus in both assays. Although the virus strains differed in the type of host cell used for manufacture, all candidate vaccine virus and target virus strains originated from an egg seed virus. Even limited passage in eggs can result in the characteristic phenotypic changes in HA, which does not revert to wild-type even after passage in eukaryotic cells. It is notable that in each set of paired results, MN titers were generally higher than HI titers, with the exception of the A/H3N2 strain in study 1. This was the only paired analysis that used a target virus in both HI and MN assays that had been grown in cells, and therefore it is possible that the manufacturing platform may have had an impact on the directionality of the relationship between the two assays. If so, it may argue for the use of homologous target virus in comparing MN and HI assays. Nevertheless, a strong positive correlation between HI and MN assays was consistently seen irrespective of laboratory vendor, study vaccine, and assay reagent, which supports the robustness of the data.

In summary, the HI and MN assays share a strong, positive correlation that indicates a high interassay association for all vaccines and strains tested. In addition, the results were highly correlated, with slopes of regression close to 1.0. Correlations were particularly high for the A/H3N2 strains in both trials. Predicted MN titers based on HI thresholds of 50% protection were consistently higher. Consistent results were observed irrespective of laboratory vendors, study vaccines, and sources of assay target virus, which supports the robustness of the data. These results support the use of the MN assay to quantify the immune response of influenza vaccines in clinical studies, particularly for the A/H3N2 strain.
